# Popular Health Methods

**Published:** 1921-04-30

**Authors:** 


### THE PUBLIC WELL-BEING.

### Popular Health Methods.

### HINTS FOR SIR ALFRED MOND.

Last week we promised to give our readers details of some of the methods that are or ought to be employed in instructing the public of all classes in matters of health. With regard to the importance insisted on by Mr. Edward Stuart who recently dealt with this subject on behalf of the League of Red Cross Societies, of the issue of official bulletins to those already engaged and interested in public health activities, vital statistics and other official information and reports are easily available in most countries, but the chief defect in England, as well as abroad, is the absence of any method of systematising these Government publications so that they present a complete and orderly survey of the national needs and conditions.

In the instruction of children by the issue of pamphlets a good deal might be done through the educational authorities. Care would have to be expended in obtaining the right kind of printed matter; and booklets on the cause and prevention of tuberculosis, the care of the teeth, bathing, fresh air, and sleep would be invaluable provided they were prepared attractively and accurately. Of course, it would be necessary to vary the material according to the ages of the children, beginning with the simplest precepts. The art work in such printed matter is of importance, and attractive drawings, especially in colours, fix the child's attention and are apt to make a permanent impression upon its mind. The Red Cross author suggests that there are unlimited possibilities in material of this kind: rhymes, songs, games, toys, alphabets, postcards may all be used to advantage. Private organisations, such as Red Cross chapters, anti-tuberculosis committees, child welfare and health centres, might co-operate in the distribution, the rôle of the health department being to originate and purchase large stocks of "literature" which could be sold at cost price to the distributor or to the children themselves. A better way still probably would be to issue them to the schools in order that they might be made use of in the ordinary school curriculum.

#### Pamphlets and Posters.

Pamphlets and circulars for adults are, of course very numerous and varied in character, but the fault which distinguishes nearly all of them is that they are too long, too technical, and too dull. The ordinary man in the street will certainly not read through twelve or sixteen pages of solid printed matter about tuberculosis (unless he is tuberculous), and women are not likely to read thirty to forty pages of printed matter on personal hygiene. We agree with the theory that it is necessary to tempt the adult public to read such publications in the same way that it is necessary to tempt them to read a motor-car catalogue, the advertisement in the magazine, or any other commercial advertisement. Communicable disease, for instance, is a dry subject to most people. Attractive drawings and pictures, good art work, directed and planned by health workers, but executed by good artists will make the people read. "A coloured picture with a small title will arouse the curiosity of the reader to see what it is about, and a picture on each page will cause him to turn page after page to see it all." The essential points on one subject can usually be condensed into a very small space. This elementary fact has been recognised by the People's League of Health, to whose admirable pamphlets we have already referred, though even in the case of these some of them at least could be made more attractive to the untrained reader and less technical in form.

What is true of pamphlets for children and adults is true also in regard to posters, in which the quality of the drawing or painting is important, for these can be very effective. On the other hand, a poor drawing is often so ludicrous that the desired effect is entirely lost. Good posters can be hung not only on the hoardings, outside post offices and public halls, or in underground railway stations, but in school class-rooms, where they can be seen month after month, thereby making a permanent impression upon the young minds. The improvement of courses in hygiene in schools is an obvious duty of health and educational authorities. Such courses can be supplemented by special lectures organised by both public and private agencies. The same is true in the case of meetings for adults, mass meetings in the evening, combining lectures with music and moving pictures, have been well attended (particularly, we believe, in the United States), when well advertised and addressed by competent and attractive speakers. Lectures can be organised in many ways: talks by local officials, talks by experts at special meetings of societies; but best of all by a full system of lectures produced and maintained by a special publicity branch of the State Department.

#### The Moving Picture.

But most of all those who are specially interested in the work of educating the public in health matters are looking to the use and development of the moving pictures as the hope of the future. Already the educational film is a commonplace in the advertisements of film companies; but so far the attempts in this direction have not been altogether satisfactory. The preparation of such films is not always in the hands of persons most suitable for the purpose, with the result that the pictures are usually superficial or inaccurate, sometimes frankly undesirable in treatment, and (a fatal defect) rarely based on a continuous or progressive plan of campaign. The value of this method, when it is definitely adopted with official sanction and brought to a pitch of perfection, is clearly found in its striking qualities, its popularity, which draws crowds to a lecture if advertised in conjunction with the cinematograph, its simplicity in size and form, which permits of

**THE PUBLIC WELL-BEING**—(*continued*).

easy transportation, and its action. Even a poor picture holds the attention of the onlookers who desire to see what is coming next.

Expositions and exhibits, showing models, placards, posters, diagrams, miniatures, practical objects, have been successfully organised, and these are valuable for short campaigns, or as permanent exhibits in large cities, especially when actual demonstration work is added, as, for example, in the case of infant care. We will conclude with a final quotation from Mr. Stuart as to the effect of a "press service" adopted by a number of health departments on the issuing of regular articles to the newspapers. "This form of publicity," he says, "has its value and is a useful supplement to health weeks and special campaigns. My opinion is that health departments should be conservative in this method in order to avoid the mistake of too much publicity; the mistake of producing too much smoke from a small fire." We venture to differ from Mr. Stuart in this opinion. There has never been, in our view, any danger of "too much publicity" from official quarters. The danger has been all the other way. What is wanted is not that health departments should be more "conservative," but more enterprising. The chief defect of these so-called publicity departments in the past has been that they are run by persons who have little knowledge of the arts of publicity and little skill in writing. The best men for this work are experienced and well-educated journalists, and certain heads of British State Departments who have lately taken such men to establish or reorganise their publicity services would be the first to acknowledge the advantages that have swiftly followed this innovation.

